# Cloning of the African indigenous cattle breed Kenyan Boran

**DOI:** 10.1111/age.12441

**Published:** 2016-04-25

**Authors:** Mingyan Yu, Charity Muteti, Moses Ogugo, William A Ritchie, Jayne Raper, Stephen Kemp

**Affiliations:** ^1^ International Livestock Research InstituteThe Joint Centre for Tropical Livestock Genetics and Health of the University of EdinburghInternational Livestock Research Institute and Scotland's Rural CollegeNairobiP.O. Box 30709‐00100Kenya; ^2^ Roslin Embryology(21) EH331QBScotlandUK; ^3^ Department of Biological SciencesHunter CollegeCity University of New YorkNew YorkNY10065USA

## Description

Kenyan Boran, an indigenous East African zebu (*Bos indicus*) breed, is kept mostly for beef production in semiarid areas of Kenya.^1^ The breed is well adapted to high ambient temperature, poor quality feed and high disease challenges compared to European exotic *Bos taurus* breeds.^2^ However, they are susceptible to African tsetse fly‐transmitted trypanosomiasis (ATT) caused by parasites (*Trypanosoma spp*.), which are also the cause of human African trypanosomiasis or sleeping sickness. The ATT is a major constraint to livestock production in sub‐Saharan Africa.^3^

It is known that serum from baboons kills both animal‐ and human‐infective African trypanosomes through serum trypanosome lytic factors (TLFs).^4^ The generation of trypanosomiasis‐resistant transgenic cattle carrying baboon‐derived TLFs may have the potential to improve livestock productivity in Kenya and Africa.^4^ As a precursor to such a study, we cloned a Kenyan Boran bull by somatic cell nuclear transfer (SCNT) using primary embryonic fibroblasts. This successful cloning represents an important first step towards the establishment of genetically modified Kenyan Boran through SCNT with genome‐modified fibroblasts.

## Methods

Boran embryonic fibroblasts were isolated from a 3‐month‐old male Kenyan Boran foetus and cultured with DMEM+10% FBS. Karyotyping was performed to confirm that the cell line had no major chromosome anomaly. Nuclei from an early passage (P6) of the fibroblasts were used for SCNT into oocytes aspirated from the ovaries of *B*. *indicus* cattle after slaughter. SCNT was performed as described,^5^ and the reconstructed blastocysts were transferred to suitable surrogate mothers. Around 273 days post‐embryo transfer, a cloned calf was born by caesarean section operation after dexamethasone induction. Ten microsatellite markers (Table S1) were used to verify the parentage of the cloned calf and its offspring.

## Results

**Figure 1:**
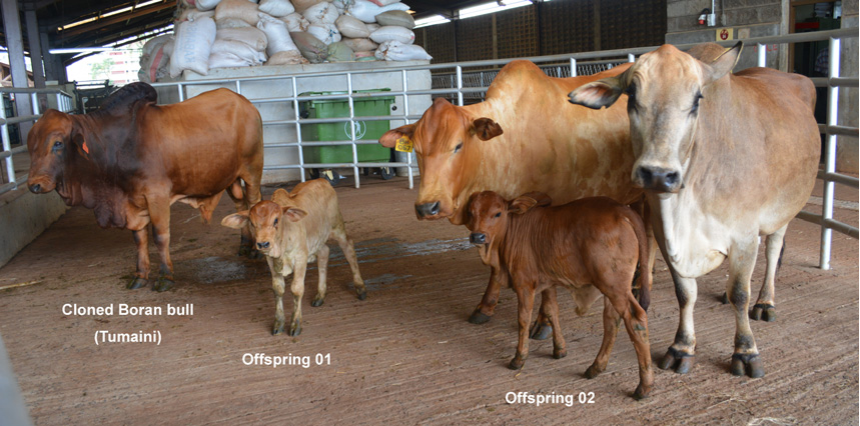
Cloned Boran bull and its offspring.

In total, three cloned embryos (60%) aborted by the second trimester, and two cloned calves (40%) were born (Table S2). Both of the born calves showed a low blood glucose level (<30 mg/dl) and difficulty in standing alone with tendon laxity at birth. The first cloned calf died apparently due to low blood glucose. The second cloned calf was supplemented with 2.5 g glucose through the jugular vein within 8 h of birth followed by abdominal injection of glucose solution (5%) when the glucose level fell below 50 mg/dl.^6^ The blood glucose level and the tendon laxity normalised after 1 week, and the calf remained healthy afterwards. Two calves were born after introducing the cloned bull to a female Boran herd (Fig. 1). Genotyping confirmed that both of the calves were offspring of the cloned bull.

## Comments

With the boom of genome editing tools, for example transcription activator‐like effector nucleases and clustered regularly interspaced short palindromic repeats/Cas9, there are unprecedented opportunities for improving livestock genetics efficiently through the introduction of superior traits between breeds by precise genome modification. The successful cloning of a Kenyan Boran bull has opened the possibility of making genetically modified Kenyan Boran with foreign genes or desired traits through genome editing at the fibroblast level followed by SCNT.

## Conflict of interest

None of the authors have any conflict of interest to declare.

## Supporting information


**Table S1** Microsatellite markers used for parentage identification of the cloned calf and its offspring

**Table S2** Summary of somatic cell nuclear transfer with Boran embryonic fibroblasts
