# Supplementary material for: Cloning of the African indigenous cattle breed Kenyan Boran
Source: Anim Genet. 2016 Apr 25;47(4):510–1. doi: 10.1111/age.12441 (PMC5074306; doi:10.1111/age.12441)
Supplement: Supplementary file 1 — Table S1 Microsatellite markers used for parentage identification of the cloned calf and its offspring [file AGE-47-510-s001.pdf]

Table S1 Microsatellite markers used for parentage identification of the cloned calf and its offspring

Marker Name	Chromosome	Primers (5'- 3')	Alleles of the donor cell line (bp)
**ILSTS098**	2	AGGAATCACTGGATAGATGC	101
		AGTGTATACTGCTTTCTCCC	105
**BM4440**	2	CCCTGGCATTCAACAAGTGT	132
		CACCCTGTTAGGAATCACTGG	136
**BMS2571**	4	CCCCAGTGATGTTCACACAG	161
		CAGCTGTCCAGCATCTGAAG	167
**RM006**	7	CTACAATATCTGGTCACTGGA	103
		GATCACCATATTTATGAGATGG	107
**INRA053**	7	AAAGTCAGATACAACTGAGTGAC	91
		AATCACCAGAAATTCACTTCACC	103
**BMS1116**	7	GAGCTTCGAGAAGGTTGGTG	144
		TCTGTGTGCATGTCTGCGT	146
**BL1043**	7	AGTGCCAAAAGGAAGCGC	111
		GACTTGACCGTTCCACCTG	114
**BMS1907**	16	AAGTGATGGGACCAGATTAGG	113
		CTCTCAAAAAGTAGTGTGTGCCT	115
**BM305**	17	ACACAATAAGAGTGTGGCATCC	103
		GTGTCCTTTTGACTCACTGTGC	122
**BMS1825**	17	CTTAACCAACACACGGTCCT	159
		CAAATACGAGAGGCTGAACAG	161
