# Supplementary material for: Cloning of the African indigenous cattle breed Kenyan Boran
Source: Anim Genet. 2016 Apr 25;47(4):510–1. doi: 10.1111/age.12441 (PMC5074306; doi:10.1111/age.12441)
Supplement: Supplementary file 2 — Table S2 Summary of somatic cell nuclear transfer with Boran embryonic fibroblasts [file AGE-47-510-s002.pdf]

Table S2 Summary of somatic cell nuclear transfer with Boran embryonic fibroblasts

No. of Oocytes	No. of Reconstructed Embryos	No. of Blastocysts	No. of Transferred Blastocysts	No. of Surrogate	No. of Pregnancy	Abortion	No. of Born Calves	No. of Survived Calves
1244	723 (58.1%[a])	85 (6.8%[a])	22	16	5	3 (60%[b])	2 (40%[b])	1 (20%[b])

[a]: the percentage is calculated against the total number of oocytes.

[b]: the percentage is calculated against the number of pregnancy.
